# Correlation analysis of serum CHI3L1, HMGB1, and CD62E detection with the prognosis of Mycoplasma pneumoniae in children

**DOI:** 10.5937/jomb0-62529

**Published:** 2026-05-22

**Authors:** Hui Yang, Tingli Zhang, Xiaoqi Zhang, Yang Xu, Jiang Xiaoan, Bo Chen

**Affiliations:** 1 Shanghai Children's Medical Center, School of Medicine, Shanghai Jiao Tong University, Department of Clinical Laboratory, Shanghai City, China; 2 Beijing Chaoyang Hospital, Capital Medical University, Department of Respiratory, Beijing, China

**Keywords:** chitinase-3-like protein 1, high mobility group protein 1, CD62E, Mycoplasma pneumoniae, correlation analysis, protein 1 sličan serumskoj hitinazi 3 (CHI3L1), protein visokomobilne grupe 1, CD62E, Mycoplasma pneumoniae, korelaciona analiza

## Abstract

**Background:**

To evaluate the clinical value of serum Chitinase-3-like protein 1 (CHI3L1), high mobility group protein 1 (HMGB1), and CD62E detection for determining the severity and prognosis of Mycoplasma pneumoniae in children.

**Methods:**

A total of 476 children with Mycoplasma pneumoniae who were diagnosed and treated at this hospital from January 2023 to March 2025 were selected as the Mycoplasma pneumoniae infection group. The healthy control group consisted of 120 children who underwent hospital examinations during the same time period. Serum levels of CHI3L1, HMGB1, and CD62E were compared between the Mycoplasma pneumoniae infection group and the healthy control group. To investigate risk factors for a poor outcome in kids infected with Mycoplasma pneumoniae, univariate and multivariate logistic regression analyses were performed. Serum levels of CHI3L1, HMGB1, and CD62E were investigated in relation to Mycoplasma pneumoniae severity and their predictive power for a poor outcome.

**Results:**

HMGB1, CD62E, and CHI3L1 serum levels were significantly greater in the Mycoplasma pneumoniae infection group than in the healthy control group (P&lt; 0.05), and they increased as Mycoplasma pneumoniae infection severity increased (P&lt; 0.05). The proportion of children with Mycoplasma pneumoniae with pleural effusion, the proportion of children with a treatment course of S7 days, the white blood cell count, the duration of antibacterial drug use, and the levels of serum CHI3L1, HMGB1, and CD62E were significantly greater in the poor prognosis group than those in the good prognosis group (P&lt; 0.05). However, no statistically significant differences were seen in CRP levels, age, or sex (P&gt; 0.05). Elevated serum CHI3L1, HMGB1, and CD62E levels were found to be independent risk factors for the prognosis of Mycoplasma pneumoniae by multivariate analysis (P&lt; 0.05). The levels of serum CHI3L1, HMGB1, and CD62E have relatively high predictive value for a poor prognosis in patients with Mycoplasma pneumoniae. The sensitivity of the combined detection of the three was 97.9%, the specificity was 90.2%, and the area under the curve was 0.785, which was significantly greater than that of the individual detection of CHI3L1, HMGB1, and CD62E (Z = 3.257, 3.429, 4.650, P&lt;0.05).

**Conclusions:**

The levels of serum CHI3L1, HMGB1, and CD62E are indicators reflecting the severity of Mycoplasma pneumoniae. The combined detection method has high efficacy in predicting the poor prognosis of Mycoplasma pneumoniae patients.

## Abstract (sr)

**Uvod:**

Cilj je bio procena kliničkog značaja određivanja nivoa CHI3L1, proteina visokomobilne grupe 1 (HMGB1) i CD62E u određivanju težine i prognoze infekcije Mycoplasma pneumoniae kod dece.

**Metode:**

Za grupu sa infekcijom je izabrano ukupno 476 dece sa Mycoplasma pneumoniae, koja je dijagnostikovana i lečena u ovoj bolnici od januara 2023. do marta 2025. godine. Kontrolnu grupu činilo je 120 zdrave dece koja su u istom periodu bila na bolničkim pregledima. Upoređeni su nivoi CHI3L1, HMGB1 i CD62E u serumu između grupe sa infekcijom i zdrave kontrolne grupe. Kako bi se istražili faktori rizika za lošu prognozu kod dece sa infekcijom Mycoplasma pneumoniae, sprovedene su univarijantna i multivarijantna logistička regresiona analiza. Istraženi su nivoi CHI3L1, HMGB1 i CD62E u vezi sa težinom bolesti i njihova prediktivna moć za lošu prognozu.

**Rezultati:**

Nivoi HMGB1, CD62E i CHI3L1 u serumu bili su značajno viši u grupi sa infekcijom Mycoplasma pneumoniae u poređenju sa zdravom kontrolnom grupom (P &lt; 0,05), a takođe su rasli sa povećanjem težine infekcije (P &lt; 0,05). Procenat dece sa pleuralnim izlivom, procenat dece sa trajanjem terapije od S7 dana, broj belih krvnih zrnaca, trajanje korišćenja antibakterijskih lekova i nivoi seruma CHI3L1, HMGB1 i CD62E u grupi sa lošom prognozom su bili značajno viši nego u grupi sa dobrom prognozom (P &lt; 0,05). Međutim, nisu uočene statistički značajne razlike u nivoima CRP-a, starosti ili polu (P&gt; 0,05). Povišeni nivoi CHI3L1, HMGB1 i CD62E u serumu pokazali su se kao nezavisni faktori rizika za prognozu infekcije Mycoplasma pneumoniae prema multivarijantnoj analizi (P&lt; 0,05). Nivoi seruma CHI3L1, HMGB1 i CD62E imaju relativno visoku prediktivnu vrednost za lošu prognozu kod pacijenata sa Mycoplasma pneumoniae. Osetljivost kombinovane detekcije sva tri markera iznosila je 97,9%, specifičnost 90,2%, a površina ispod krive (AUC) 0,785, što je značajno više nego kod pojedinačne detekcije CHI3L1, HMGB1 i CD62E (Z = 3,257, 3,429, 4,650, P&lt;0,05).

**Zaključak:**

Nivoi seruma CHI3L1, HMGB1 i CD62E predstavljaju indikatore koji odražavaju težinu infekcije Mycoplasma pneumoniae. Metod kombinovane detekcije ima visoku efikasnost u predviđanju loše prognoze kod pacijenata sa Mycoplasma pneumoniae.

## Introduction

Mycoplasma pneumoniae is a frequent cause of respiratory infection in children, with a clinical incidence rate of 10% to 20% and a peak of 30% during the epidemic season [1][2][3]. The prevalent age group is 5-14 years. It often presents with cough, fever, and shortness of breath. In severe cases, symptoms such as nausea, vomiting, and chest pain may occur, and dysfunction of other organs may even develop [4]. The condition of critically ill children progresses rapidly, and complications such as respiratory distress syndrome, shock, and multiple organ failure occur in a short period of time, seriously threatening their safety [5][6][7].

Although indicators such as imaging, blood, and sputum cultures can help determine the child's condition, they lag in accurately reflecting it. They cannot quantitatively reflect it in real time [8]. Serological indicators exhibit real-time, quantitative, and repeatable detection characteristics and have broad application prospects [9]. Although the pathogenesis of *Mycoplasma pneumoniae* remains unclear, immune disorders and excessive inflammatory responses play significant roles in the occurrence and development of this disease [10][11][12]. Chitinase-3-like protein 1 (CHI3L1) is involved in airway remodeling and hyperreactivity in pneumonia and plays an essential role in determining disease conditions and patient prognosis. Widely distributed in eukaryotic cells, high mobility group protein 1 (HMGB1) is released from inflammatory cells outside of the cell to contribute to the body's inflammatory response [13]. High mobility group protein 1 (HMGB1) is widely distributed in eukaryotic cells and is released from inflammatory cells outside of the cell to contribute to the body's inflammatory response [14]. CD62E is a crucial marker of early inflammatory responses and a member of the adhesion molecule family that facilitates white blood cell adhesion and migration [15].

In this study, serum levels of CHI3L1, HMGB1, and CD62E were measured together to determine their clinical utility in predicting the course and outcome of pediatric Mycoplasma pneumoniae.

## Materials and methods

### General information

A total of 476 children with *Mycoplasma pneumoniae* who were diagnosed and treated in our hospital from January 2023 to March 2025, including 260 boys and 216 girls, were included in the *Mycoplasma pneumoniae* infection group. The ages ranged from 5 to 11 years, with an average of 7.93± 1.37 years. Based on severity, the patients were split into 196 severe cases (severe group) and 280 moderate cases (mild group).

The diagnostic criteria for *Mycoplasma pneumoniae* in children are as follows: (1) the child is in the preschool or school-age period; (2) the child has fever and corresponding respiratory symptoms; (3) X-ray images reveal patchy or patchy infiltrative changes or changes in interstitial pneumonia; (4) a positive test for *Mycoplasma pneumoniae* antibodies in the serum; (5) a positive nucleic acid test of the throat swab. If any of the above conditions (1) to (3) are met and any one of (4) or (5) is also met, the diagnosis of *Mycoplasma pneumoniae* will be made. In addition to meeting the above diagnostic criteria, severe *Mycoplasma pneumoniae* is associated with the following conditions: (1) digestive tract symptoms such as diarrhea, nausea and vomiting; (2) a significant increase in pulse and heart rate; (3) neurological symptoms such as drowsiness or irritability; and (4) the presence of exudative shadows and other manifestations on chest X-ray. Another 120 kids, 58 of whom were girls and 62 of whom were boys, who received medical examinations at our facility during that time, were chosen as the healthy control group. The average age ranged from 5 to 11 years, with a mean of 7.96±1.33 years.

The inclusion criterion was as follows: the *Mycoplasma pneumoniae* infection group was diagnosed with *Mycoplasma pneumoniae* infection by nucleic acid testing or mycoplasma culture, and the healthy control group consisted of healthy children. All patients were aged 5-11 years, and the clinical data were complete. The exclusion criteria for patients were as follows: other types of infections, such as bacterial or viral infections; had recently received immunotherapy or hormone therapy; had combined blood and immune system diseases; had combined blood and malignant tumors; had congenital respiratory diseases; or had intellectual disabilities or mental disorders.

Each research participant signed an informed consent form, and the hospital ethics committee approved this study.

### Treatment and prognosis grouping

Children in the* Mycoplasma pneumoniae* infection group received basic treatments, including cough suppressants, expectorants, anti-infectives, asthma relief, and fever reduction. For patients with a high fever that does not resolve and elevated lactate dehydrogenase and C-reactive protein (CRP) levels, hormone therapy was administered. Patients with severe extrapulmonary complications should be treated with gamma globulin. Children who develop unilateral clear lung disease, within three weeks of treatment, patients with organized pneumonia, pulmonary fibrosis, or obliterate bronchitis are categorized as having a bad prognosis. In this study, 82 patients were included in the poor-prognosis group, and the remaining 394 in the good-prognosis group.

### Collection and testing of blood samples

When research subjects were admitted to the hospital or underwent outpatient physical examinations, approximately 5 mL of venous blood was collected from the elbow vein. The blood was placed at room temperature and centrifuged, and the supernatant was stored at -80°C for testing. The white blood cell count was measured using an automatic blood cell counter (Hitachi 7600). CRP was determined by a dry immunofluorescence analyzer (i-CHROMA, Aikemei). Using an enzyme-linked immunosorbent test, the levels of serum CHI3L1, HMGB1, and CD62E were measured. All reagent kits were produced by Beijing Bell Bioengineering Co., Ltd., and were used in strict accordance with the instructions provided with each kit.

### Observation indicators

Serum levels of CHI3L1, HMGB1, and CD62E were compared between the Mycoplasma pneumoniae infection group and the healthy control group. Factors associated with a poor prognosis in patients with Mycoplasma pneumoniae were examined using univariate and multivariate analyses. The correlations between Mycoplasma pneumoniae severity and serum CHI3L1, HMGB1, and CD62E levels, as well as their diagnostic efficacy in predicting poor prognosis, were investigated.

### Statistical analysis

SPSS 20.0 was used to analyze the data. The measurement data, which follow a normal distribution, are reported as x̄±s, and a t-test was used to compare the two groups. Count data were expressed as percentages or counts, and the χ^2^ test was used to compare groups. Multivariate logistic regression was used to analyze the risk factors for the prognosis of children with *Mycoplasma pneumoniae*. The predictive efficacy of each index was analyzed via receiver operating characteristic (ROC) curves.

## Results

### Comparison of serum CHI3L1, HMGB1 and CD62E levels between the two groups

The Mycoplasma pneumoniae-infected group had significantly greater serum levels of CHI3L1, HMGB1, and CD62E than the healthy control group (P<0.05).

Children with Mycoplasma pneumoniae infection had significantly higher serum levels of CHI3L1, HMGB1, and CD62E than those in the healthy control group. This trend is not only reflected in the overall comparison between the infected and healthy groups but is also closely related to the severity of the disease in children. Within the infection group, as the severity of Mycoplasma pneumoniae increased, the levels of these three serum markers also showed a consistent, rising trend. These findings collectively indicate that serum CHI3L1, HMGB1, and CD62E are activated in the pathologic process of Mycoplasma pneumoniae in children, and that changes in their levels can effectively reflect the body's inflammatory state and the severity of the disease (Table 1).

**Table 1:** Comparison of serum CHI3L1, HMGB1, and CD62E levels between the two groups (x̄ ± s).

Group	n	CHI3L1 (ng/mL)	HMGBl (×10^-6^ ng/mL)	CD62E (μg/L)
Healthy control group	120	25.61±3.20	284.72±36.61	0.69±0.14
Mycoplasma pneumoniae<br>infection group	476	47.28±272	1036.58±97.40	3.38±0.59
t		51.633	95.215	69.106
P		<0.001	<0.001	<0.001

### Comparison of serum CHI3L1, HMGB1, and CD62E levels between the severe group and the mild group

The severe group had significantly greater serum levels of CD62E, HMGB1, and CHI3L1 than the mild group (P<0.05).

As the children's health worsened from mild to severe, the expression levels of CHI3L1, HMGB1, and CD62E did not remain constant but instead exhibited a notable upward trend. The more severe the child's condition, the greater the increase in the concentrations of these three indicators in the child's body. These findings strongly indicate that the serum concentrations of CHI3L1, HMGB1, and CD62E can sensitively reflect the intensity of the inflammatory response and the degree of pathological damage caused by mycoplasma infection in the body, and that changes in their concentrations can be used as critical biological indicators for evaluating the severity of mycoplasma pneumonia in children (Table 2).

**Table 2:** Comparison of serum CHI3L1, HMGB1, and CD62E levels between severe and mild groups (x̄ ± s).

Group	n	CHI3L1 (ng/mL)	HMGBl (×10^-6^ng/mL)	CD62E (μg/L)
Severe group	196	49.80±1.73	1122.36±81.86	3.84±0.47
Mild group	280	45.44±1.74	976.54±52.06	3.06±0.31
t		19.847	15.577	14.381
P		<0.001	<0.001	<0.001

### Univariate analysis of the prognosis of Mycoplasma pneumoniae

The proportion of patients with pleural effusion, white blood cell count, length of antibacterial drug use, the percentage of patients with a treatment course of ≥7 days, as well as the levels of serum CHI3L1, HMGB1, and CD62E, were significantly greater in the group with a poor prognosis than in the group with a good prognosis (P<0.05). Age, sex, CRP level, and length of glucocorticoid use did not differ statistically significantly between the two groups (P>0.05).

The preliminary analysis results suggest that pleural effusion, a longer treatment course, a higher white blood cell count, a longer duration of antibiotic use, and significantly elevated levels of serum CHI3L1, HMGB1, and CD62E may all be associated with a poor prognosis in children with *Mycoplasma pneumoniae* (Table 3).

**Table 3:** Univariate analysis of prognosis for *Mycoplasma pneumoniae* [n (%) or x̄ ± s].

Indicator	n	Poor prognosis<br>group (n=82)	Good prognosis<br>group (n = 197)	x^2^/t	P
Gender				0.520	0.461
Male	260	50 (60.98)	210 (53.30)		
Female	216	32 (39.02)	184 (46.70)		
Age (years)	476	7.88±1.36	7.94±1.38	0.243	0.814
pleural effusion				9.793	0.005
yes	214	56 (68.29)	158 (40.10)		
no	262	26 (31.71)	236 (59.90)		
Course of treatment (d)				8.501	0.007
≥7	188	50 (60.98)	138 (35.03)		
≤7	288	32 (39.02)	256 (64.97)		
White blood cell count (×10^9^/L)	476	9.80±2.03	8.53±1.98	8.856	<0.001
CRP(mg/L)	476	34.09±6.08	32.96±6.48	1.034	0.307
Duration of use of antibiotics (d)	476	12.08±2.91	9.55±2.88	5.121	<0.001
Duration of glucocorticoid use (d)	476	9.37±1.82	8.94±2.42	1.047	0.290
CHI3L1 (ng/mL)	476	50.22±2.13	46.64±2.41	8.856	<0.001
HMGB1 (×10^-6^ ng/mL)	476	1159.11±109.37	1011.06±72.47	8.308	<0.001
CD62E (μg/L)	476	3.98±0.77	3.25±0.45	6.071	<0.001

### Multivariate logistic analysis of the prognosis of Mycoplasma pneumoniae

The factors with statistical significance in the univariate analysis were included, and a multivariate logistic regression was conducted based on whether a poor prognosis occurred. The prognosis of Mycoplasma pneumoniae was found to be independently influenced by elevated levels of serum CHI3L1, HMGB1, and CD62E (P<0.01). In contrast, disease duration, pleural effusion, treatment course, white blood cell count, and duration of antibiotic use were not independent risk factors for *Mycoplasma pneumoniae* (P>0.05) (Table 4).

**Table 4:** Multivariate logistic analysis of prognosis of Mycoplasma pneumoniae.

Item	β	SE	Wald	P	OR	95% CI
Pleural effusion	0.142	1.169	0.019	0.891	1.164	0.111~11.405
Course of treatment	1.336	1.133	1.395	0.231	3.797	0.417~34.767
White blood cell count	0.519	0.295	3.138	0.070	1.679	0.949~2.961
Duration of use of antibiotics	0.378	0.195	3.811	0.054	1.458	0.992~2.112
CHI3L1	0.728	0.208	12.456	<0.001	2.068	1.383~3.081
HMGB1	0.033	0.001	16.652	<0.001	1.033	1.019~1.048
CD62E	4.379	1.080	16.214	<0.001	79.558	9.453~669.713

### Predictive efficacy of serum CHI3L1, HMGB1, and CD62E detection for poor prognosis in patients with Mycoplasma pneumoniae

The poor prognosis of patients with Mycoplasma pneumoniae can be reliably predicted by serum levels of CHI3L1, HMGB1, and CD62E. Binary logistic regression analysis was conducted on the above indicators to determine whether *M. pneumoniae* pneumonia is associated with a poor prognosis. The equation Y=0.65xXCHI3L1+0.03xXHMGB1+4.84xXE selectin -82.59 was obtained. The combined detection index was calculated. The sensitivity of the combined detection of the three was 97.9%, and the specificity was 90.2%. The area under the curve (AUC) was 0.985, which was significantly greater than that of CHI3L1, HMGB1, and CD62E detected separately (Z = 3.257, 3.429, and 4.650, P<0.01). In contrast, there was no statistically significant difference in the AUC among the three indicators (P>0.05) (Table 5).

**Table 5:** Predictive efficacy of serum CHI3L1, HMGB1, and CD62E levels for poor prognosis of Mycoplasma pneumoniae.

Indicator	Truncation value	Sensitivity (%)	Specificity (%)	AUC	95% CI
CHI3L1	49.6 ng/mL	75.9	86.6	0.873	0.823~0.913
HMGBl	1098.8 ng/mL	78.3	91.2	0.860	0.810~0.901
CD62E	3.69 μg/L	70.0	92.2	0.807	0.741~0.856
CHI3L1+HMGB1<br>CD62E	-	97.9	90.2	0.986	0.950~0.998

## Discussion

Pneumonia is a common respiratory disease caused mainly by inflammation of the lungs in the terminal airways, interstitial spaces, and alveoli [16]. This disease is prevalent among children, with bronchopneumonia having the highest incidence rate. It seriously endangers children's health, and its fatality rate is the leading cause of infectious diseases [17]. *Mycoplasma pneumoniae* is a self-limiting pneumonia. If the condition is not taken seriously enough, it can lead to extrapulmonary complications, with serious consequences for the child [18][19][20]. Although lung examination and sputum culture help determine the condition and prognosis of pediatric patients, they cannot reflect changes in the internal environment or inflammatory factors, and their ability to accurately assess condition and prognosis is limited [21]. This study revealed that the poor prognosis of children with *Mycoplasma pneumoniae* was associated with pleural effusion, treatment duration, white blood cell count, and levels of CHI3L1, HMGB1, and CD62E, but was not correlated with age, sex, or CRP [22][23].

The serum CHI3L1 level in the *Mycoplasma pneumoniae* infection group was significantly greater than that in the healthy control group [24]. Moreover, as disease severity increased, serum CHI3L1 levels in children with *Mycoplasma pneumoniae* increased significantly, indicating that CHI3L1 is involved in the pathogenesis of *Mycoplasma pneumoniae*. CHI3L1 is a novel inflammatory factor and a secreted glycoprotein that is mainly secreted by activated neutrophils and macrophages [25]. It plays a significant role in the occurrence and development of lung diseases. When lung inflammation occurs, the serum level of CHI3L1 also increases significantly and has been used as an indicator of the airway inflammatory response and lung severity [26]. CHI3L1 has been confirmed in basic research to promote the proliferation and migration of bronchial smooth muscle cells in pneumonia, cause airway damage in children, and ultimately affect lung function [27]. Additionally, CHI3L1 plays a significant role in controlling pulmonary inflammatory responses, airway remodeling, and airway hyperresponsiveness [28]. When the serum CHI3L1 concentration was 49.3 ng/mL, the sensitivity for predicting poor prognosis in children with *Mycoplasma pneumoniae* was 75.9%, the specificity was 86.6%, and the AUC was 0.873, indicating that the serum CHI3L1 concentration is an important indicator reflecting the prognosis of children with *Mycoplasma pneumoniae* [29]. Therefore, for children with severe *Mycoplasma pneumoniae* infection, when the serum CHI3L1 concentration is >49.3 ng/mL, additional treatment measures are needed to improve their prognosis, which warrants further clarification in future studies [30].

HMGB1 is widely distributed in the heart, brain, lungs, and lymphoid tissues [31]. Activated macrophages, monocytes, and natural killer cells mainly secrete it. After binding to the corresponding receptors, it produces corresponding biological effects. Studies have shown that elevated serum HMGB1 levels promote cytokine production and are involved in the pathophysiological process of lung inflammatory injury [32]. This study revealed that the serum HMGB1 level in the *Mycoplasma pneumoniae* infection group was higher than that in the healthy control group, and that the serum HMGB1 level in the severe group was significantly higher than that in the mild group [33]. These findings indicate that HMGB1 is associated with the occurrence and progression of Mycoplasma pneumoniae and is an indicator of pneumonia severity. HMGB1 plays a significant role in the occurrence of acute lung injury [34]. After HMGB1 was blocked, lung injury was significantly relieved. After HMGB1 binds to the surface receptors of endothelial cells and immune cells, it disrupts the epithelial barrier and releases various cells, thereby aggravating the disease [35]. The serum HMGB1 concentration was 1,098.8 ng/mL. The sensitivity for predicting poor prognosis in children with *Mycoplasma pneumoniae* was 78.3%, the specificity was 91.2%, and the AUC was 0.860, indicating that serum HMGB1 has high efficacy in predicting poor prognosis in these patients and is an important prognostic indicator for *Mycoplasma pneumoniae*.

This study revealed that the serum CD62E level in the *Mycoplasma pneumoniae* infection group was significantly higher than that in the healthy control group and increased with the severity of Mycoplasma pneumoniae infection, indicating that the occurrence and progression of Mycoplasma pneumoniae are associated with CD62E levels [36]. CD62E is a member of the adhesion molecule superfamily and is mainly secreted by vascular endothelial cells. The inflammatory response releases a variety of cytokines, stimulating vascular endothelial cells to secrete CD62E and exerting corresponding biological effects through interactions between white blood cells and endothelial cells, such as regulating cell proliferation, and is involved in the occurrence and development of *Mycoplasma pneumoniae* [37]. This study also revealed that the serum CD62E level in the poor-prognosis group was significantly higher than that in the good-prognosis group. When the CD62E concentration was 3.69 μg/L, the sensitivity for predicting poor prognosis was 70.0%, the specificity was 92.2%, and the AUC was 0.807, indicating that the serum CD62E concentration has high efficacy in predicting poor prognosis in patients with *Mycoplasma pneumoniae*. This study revealed that the combined detection of serum CH 13 L1, HMGB1, and CD62E levels was significantly more effective at predicting poor prognosis in Mycoplasma pneumoniae than the detection of individual markers. Its sensitivity was 97.9% and its specificity was 90.2%, indicating some complementarity among the three. The specific interaction mechanism needs further study.

## Conclusion

Serum levels of CHI3L1, HMGB1, and CD62E are markers of Mycoplasma pneumoniae severity. The combined detection method has high efficacy in predicting the poor prognosis of *Mycoplasma pneumoniae* patients.

## Dodatak

### Conflict of interest statement

All the authors declare that they have no conflict of interest in this work.
